# Prevention of House Dust Mite Induced Allergic Airways Disease in Mice through Immune Tolerance

**DOI:** 10.1371/journal.pone.0022320

**Published:** 2011-07-26

**Authors:** Ana Agua-Doce, Luis Graca

**Affiliations:** 1 Instituto de Medicina Molecular, Faculty of Medicine, University of Lisbon, Lisbon, Portugal; 2 Instituto Gulbenkian de Ciência, Oeiras, Portugal; University Paris Sud, France

## Abstract

Allergic airways disease is a consequence of a Th2 response to an allergen leading to a series of manifestations such as production of allergen-specific IgE, inflammatory infiltrates in the airways, and airway hyper-reactivity (AHR). Several strategies have been reported for tolerance induction to allergens leading to protection from allergic airways disease. We now show that CD4 blockade at the time of house dust mite sensitization induces antigen-specific tolerance in mice. Tolerance induction is robust enough to be effective in pre-sensitized animals, even in those where AHR was pre-established. Tolerant mice are protected from airways eosinophilia, Th2 lung infiltration, and AHR. Furthermore, anti-CD4 treated mice remain immune competent to mount immune responses, including Th2, to unrelated antigens. Our findings, therefore, describe a strategy for tolerance induction potentially applicable to other immunogenic proteins besides allergens.

## Introduction

The control of deleterious immune responses causing diseases, such as allergy, autoimmunity and transplant rejection, has been one of the main objectives of immunologists. Moreover, the global prevalence of this type of diseases has been steadily increasing.

Several strategies have been recently described to induce tolerance to allergens thus preventing allergic airways disease [1], [2], [3], [4]. In brief, they can rely on the induction of dendritic cell (DC) populations or regulatory T cells (Treg) able to control pathologic T cell clones, in a process where IL-10 and TGF-β can participate [1], [5], [6], [7], [8], [9], [10]. In addition, disease prevention may be achieved by skewing the immune response from a Th2 to a Th1 phenotype [11].

In fact, the realization of the critical importance of T cells in the pathogenesis of allergic airways disease was well demonstrated by studies where anti-CD4 monoclonal antibodies (MAbs) causing the depletion of this T cell subset could prevent the disease in mice [12]. Such pre-clinical studies with CD4 T cell depletion provided the rationale for clinical trials with depleting anti-CD4 MAbs where the short-term benefit observed was probably associated with transient immune suppression [13]. As a consequence, the interest has shifted towards MAbs capable of blocking molecular interactions but without leading to direct cell lysis.

Some reports have shown prevention of allergic airways disease following the blockade of T cell co-stimulatory or co-receptor molecules with non-depleting MAbs, but it remains unclear whether long-term antigen-specific tolerance is achieved or what are the mechanisms involved [14], [15], [16], [17]. We now describe CD4 blockade at the time of exposure with a model antigen, ovalbumin (OVA), or a clinically relevant allergen, house dust mite (HDM), can induce antigen-specific tolerance and protection from allergic airways disease. The mechanism leading to antigen-specific tolerance without affecting protective immune responses (including Th2-type responses) to additional antigens is independent of a switch between a Th2-type and Th1-type immune response. Since CD4 blockade is achieved with a non-depleting MAb, T cells not activated by the antigen remain unaffected to mount protective immune responses towards unrelated antigens at a later time.

Tolerance induction by CD4 blockade is robust enough to be effective in pre-sensitized animals and even in animals where AHR was previously established. The tolerant mice show protection from allergic manifestations elicited by intranasal exposure to the antigen: they do not develop airways eosinophilia, goblet cell hyperplasia, production of Th2 cytokines in the lung, production of antigen-specific IgE or IgG1, and, importantly, do not develop airway hyperreactivity (AHR) in response to inhaled methacholine (MCh).

## Results

### Co-receptor blockade with non-depleting anti-CD4 MAb prevents allergic sensitization in mice

Using a well established murine model of allergic airways disease we sought to determine if non-depleting MAbs targeting the T cell co-receptor molecule CD4 were effective in preventing allergic sensitization with HDM or a model antigen (OVA).

**Figure 1:**
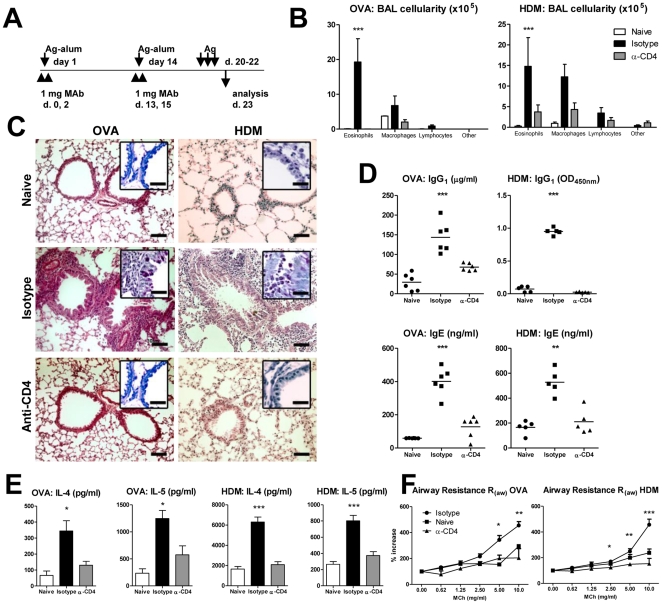
Prevention of allergic sensitization with anti-CD4 MAb. (A) Female BALB/c mice were sensitized with 20 µg OVA-alum or 50 µg HDM-Alum i.p. and challenged with 50 µg OVA or HDM in saline i.n. on the indicated days. Some animals were treated with 1 mg anti-CD4 or an isotype control i.p. as shown. Naive mice, not subjected to any intervention, were also studied as a control group. (B) Cellular composition of the BAL. Animals treated with anti-CD4 have less eosinophils in the BAL (n = 6, *** *P*<0.001). (C) Histological sections of lung tissue were stained with hematoxilin/eosin and PAS (inset). Anti-CD4-treated mice have reduced inflammatory infiltrate and goblet cell hyperplasia, to levels similar to naive controls. Bars represent 10 µm (2.5 µm in the inset). (D) Quantification of serum OVA- and HDM-specific IgG1 and IgE. Anti-CD4 MAb treated mice show a significant reduction of the Th2-driven immunoglobulins (n = 6, *** *P*<0.001 and ** *P*<0.01 as indicated). (E) The Th2 cytokines IL-4 and IL-5 were down to basal levels in lung homogenates of anti-CD4 treated mice (n = 6, * *P*<0.05 and *** *P*<0.001). (F) Invasive measurement of respiratory mechanics shows that animals treated with anti-CD4 MAbs had reduced airway resistance to increasing doses of inhaled MCh, when compared with sensitized control animals (n = 8). Data (B–F) are representative of at least three independent experiments.

BALB/c mice were sensitized with two i.p. injections of OVA-alum or HDM-alum on days 1 and 14, and challenged with 50 µg OVA or HDM i.n. on days 20, 21 and 22 (Figure 1A). Experimental groups were treated with 1 mg i.p. of anti-CD4 or an isotype control on the days before and after each immunization, and sacrificed 24 hours following the last intranasal challenge.

Mice treated with anti-CD4 had a marked reduction in BAL eosinophils when compared with sensitized animals, to levels similar to naïve animals or animals sensitized in the absence of the antigen (Figure 1B, and Figure S1). The absence of goblet cell hyperplasia and inflammatory infiltrate in the airways of anti-CD4 treated mice was confirmed by histology (Figure 1C). Furthermore, anti-CD4 treatment prevented effective generation of Th2-driven OVA- and HDM-specific IgG1 and IgE (Figure 1D). We could not detect Th1-driven antigen-specific IgG2a in any animal (not shown). Animals treated with anti-CD4 showed a marked reduction of IL-4 and IL-5 in lung homogenates to levels similar to naive animals (Figure 1E). Importantly, we found no evidence for Th1 or Th17 deviation (as inferred by levels of IFNγ or IL-17), nor increased levels of the immune-regulatory cytokine IL-10 (not shown). Cytokines in BAL were similar to lung homogenates (not shown).

To study the functional impact of the treatment we assessed AHR in response to increasing doses of inhaled MCh. Our data show that anti-CD4 treatment prevented AHR (Figure 1F and Figure S2).

### The tolerance state is maintained following clearance of the MAb

**Figure 2 pone-0022320-g002:**
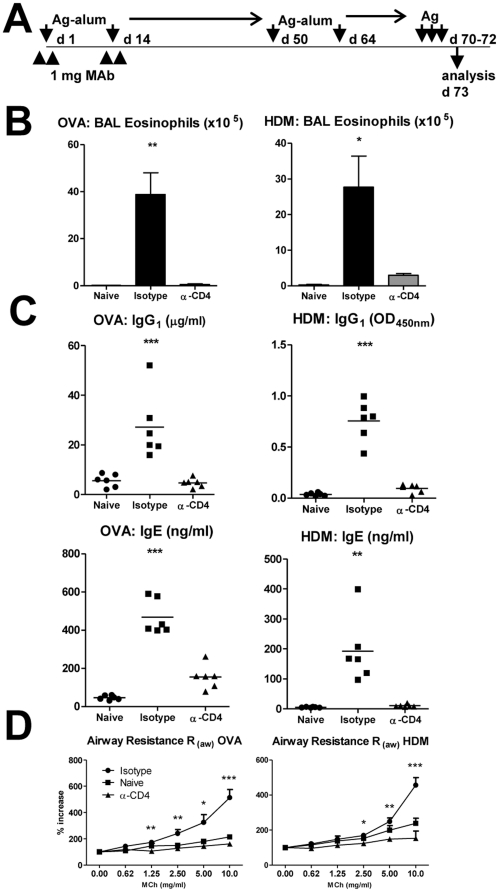
Tolerized mice resist subsequent sensitization and challenge. (A) BALB/c mice were initially sensitized with OVA or HDM under the cover of anti-CD4 as described in Figure 1. Those mice were sensitized with the same antigens on days 50 and 64, and subsequently challenged i.n. (B) Animals trated with anti-CD4 were protected from BAL eosinophilia (n = 6, ** *P*<0.01 for OVA; * *P*<0.05 for HDM). (C) CD4-blockade prevented production of IgG1 and IgE in subsequent sensitizations (n = 6, *** *P*<0.001 and ** *P*<0.01 as indicated). (D) Tolerance to OVA or HDM prevented AHR to inhaled MCh (n = 6). Data are representative of three independent experiments.

We then studied whether tolerance induction with non-depleting anti-CD4 would protect the animals from subsequent exposure to the same antigens, at a time the therapeutic MAb had been cleared. For this purpose treated mice with anti-CD4 at the time of initial sensitization with OVA or HDM, and the same mice were again immunized with the same antigens 50 days following the initial treatment (Figure 2A).

Sensitization with OVA-alum or HDM-alum did not lead to airways eosinophilia in mice previously exposed to the same antigens under the cover of non-depleting anti-CD4 (Figure 2B and Figure S1). Furthermore, the treated mice also failed to produce antigen-specific IgG1 and IgE to OVA-alum and HDM-alum (Figure 2C). And importantly, AHR to increased concentrations of inhaled MCh was also absent in anti-CD4 treated mice (Figure 2D and Figure S2).

We could therefore conclude that a short course of anti-CD4 was leading to long-term effects. Although the MAb we used (clone YTS177) is known to have a non-depleting isotype [18], and we confirmed anti-CD4 treatment was not directly leading to T cell lysis (not shown), we had to confirm the treated mice remained immune competent.

### Tolerant mice remain immunocompetent

In order to study the antigen-specificity of tolerance induction, we used a second unrelated antigen: β-lactoglobulin (β-LG). We compared immune responses to OVA and β-LG, since these are two defined antigens with similar characteristics, while HDM is a complex protein extract containing many distinct antigens.

**Figure 3 pone-0022320-g003:**
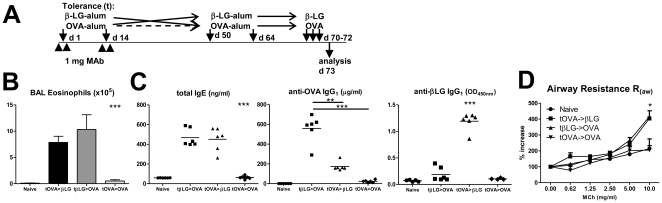
Mice treated with anti-CD4 are competent to respond to unrelated antigens. (A) Mice initially tolerized to OVA or β-LG (as described in previous figures) were sensitized i.p. with a different antigen at days 50 and 64, and challenged i.n. with the same antigen used at day 50. (B) Only animals tolerized to the same antigen used for sensitization at day 50 were protected from BAL eosinophilia (n = 6, *** *P*<0.001). (C) Tolerance induction to OVA did not prevent subsequent production of β-LG-driven IgE or IgG1, conversely, tolerance to β-LG did not hamper the generation of OVA-specific IgG1 or IgE (n = 6, *** *P*<0.001). (D) AHR in response to MCh was observed in animals tolerized to an antigen different from the one used for subsequent sensitization (tOVA >β-LG and tβ-LG >OVA) (n = 8, * *P*<0.05 at 10 mg/ml MCh). Data are representative of two independent experiments.

BALB/c mice were treated with non-depleting anti-CD4 MAb together with OVA-alum (tOVA) or β-LG-alum (tβ-LG) in order to establish immune tolerance to those antigens (Figure 3A). At day 50 the animals were immunized with the same antigen used at the time of tolerization (day 0) or with the second unrelated antigen. All mice were subsequently challenged i.n. with the same antigen used at day 50. All animals remained protected from mounting airways inflammation in response to the antigen used for tolerization, but fully competent to undergo a Th2 response to the second antigen leading to airways eosinophilia, production of IgE and IgG1, and AHR (Figure 3B–D and Figure S2). These results suggest that CD4-blockade affects specifically the T cells that are being activated at the time of treatment, and sparing non-activated cells, thus leading to antigen specific tolerance where immune responses against different antigens are not suppressed.

### Tolerance can be achieved in sensitized mice

**Figure 4 pone-0022320-g004:**
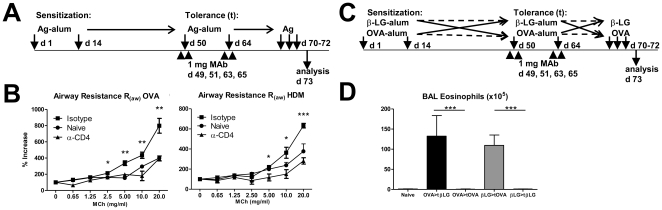
Tolerogenic effect of anti-CD4 treatment is antigen-specific and effective in sensitized animals. (A) BALB/c mice sensitized with OVA-alum or HDM-alum were tolerized to the same antigens on days 50 and 64, and challenged i.n. with the same antigens. (B) Sensitized mice subsequently treated with OVA or HDM under the cover of anti-CD4 showed protection from AHR (n = 8 for OVA, n = 6 to HDM). Data are representative of two independent experiments. (C) Mice were initially sensitized with OVA-alum or β-LG-alum, and tolerized to the same or a different antigen on days 50 and 64. All mice were challenged i.n. with the same antigen used for initial sensitization. (D) Mice treated with a different antigen together with anti-CD4 did not show reduced BAL eosinophilia (OVA >tβ-LG and β-LG >tOVA) while treatment with anti-CD4 and the same antigen used for sensitization showed a significant reduction of BAL eosinophilia (OVA>tOVA and β-LG >tβ-LG; n = 6, *** *P*<0.001). Data are representative of two independent experiments.

To assess whether tolerance can be induced in pre-sensitized mice, BALB/c mice sensitized with OVA or HDM were treated with the same antigen under the cover of anti-CD4 50 days following initial sensitization (Figure 4A). For consistency with previous experiments we maintained the tolerance-inducing regime as two administrations of antigen-alum+anti-CD4 two weeks apart.

We found, both OVA- and HDM-sensitized mice treated with anti-CD4 were prevented from AHR, maintaining normal airway response to increased concentrations of inhaled MCh (Figure 4B and Figure S2). We confirmed the efficient sensitization of all groups of immunized animals by the presence of antigen-specific IgG1 and IgE antibodies in sera (not shown), although the mice had not been exposed to prior airway inflammation. As a consequence, the protection from AHR is effective in spite of high titres of antigen-specific immunoglobulins – possibly representing an impact on the late-phase response, and dissociation between high IgE and AHR.

We then assessed whether tolerance induction in pre-sensitized mice remained antigen-specific. BALB/c mice sensitized with OVA-alum or β-LG-alum were treated with anti-CD4 MAb, 50 days following the initial intervention, in the presence of either the initial (OVA or β-LG) or a different antigen (β-LG or OVA, respectively; Figure 4C). Sensitized mice were protected from airway eosinophilia when treated with anti-CD4 in the presence of the same antigen used for sensitization (OVA >tOVA and β-LG >tβ-LG, Figure 4D). The protective effect was, therefore, not due to the persistence of the therapeutic antibody in circulation at the time of intranasal exposure to the antigen since mice immunized with a different antigens not present during anti-CD4 treatment (and therefore with equivalent doses of circulating anti-CD4 at the time of challenge) were not protected (OVA >tβ-LG and β-LG >tOVA). The observation that animals receiving the antibody treatment together with a different antigen than used for immunization develop inflammatory changes similar to untreated control animals, or animals exposed to alum in the absence of the antigen (Figure S1), are consistent with the antigen-specificity of the tolerance state described above.

### Mice exposed to allergic airways disease can be protected from AHR following anti-CD4 treatment

**Figure 5:**
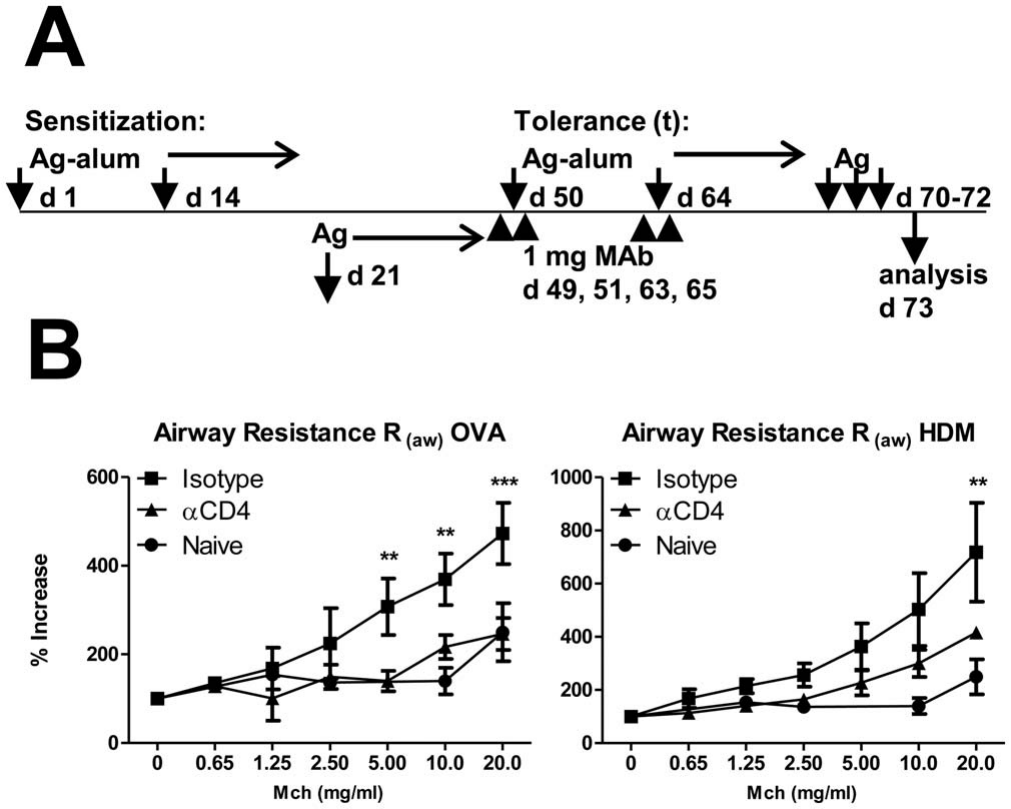
Protection from AHR in animals previously exposed to airways inflammation. (A) Balb/c mice sensitized and challenged i.n. with HDM-alum or OVA-alum were tolerized to the same antigens on days 50 and 64, and challenged i.n. with the same antigens. (B) The animals treated with HDM or OVA under the cover of anti-CD4 were protected from AHR in response to inhaled MCh (n = 6, ** *P*<0.01 or *** *P*<0.001 as indicated). Data are representative of two independent experiments.

However, it is understood that the onset of inflammation in the airways becomes a significant hurdle for immune modulation leading to tolerance. Therefore, we investigated whether mice sensitized to OVA or HDM and exposed to the antigen i.n. could benefit from subsequent anti-CD4 treatment (Figure 5A). A single i.n. challenge with antigen in sensitized mice was sufficient to induce AHR (Figure S3).

We found that administration of HDM under the cover of non-depleting anti-CD4 30 days following induction of allergic airways disease was effective in preventing AHR following subsequent challenge with the same antigen (Figure 5B). We repeated the same studies with OVA with similar results (Figure 5B and Figure S2).

## Discussion

Our data shows that CD4 blockade is effective in inducing antigen-specific tolerance to a clinically relevant allergen (HDM), thus preventing the manifestations of allergic airways disease following intranasal allergen challenge: Th2 and eosinophilic infiltrate of the airways, goblet cell hyperplasia, and AHR. The tolerogenic treatment is not only effective in preventing the disease in naive animals, but also confers considerable protection to mice previously sensitized with the allergen. Although in different experiments we tested mice with different ages, we could not find an age-related difference in their response to induction of allergic airways disease (Figure S4).

It should be noted that the use of tolerogenic MAbs in transplantation, with the same objective of preventing an inflammatory response to non-self antigens, have resulted in a different outcome from what we have observed. In transplantation, tolerance induction in sensitized animals has been difficult to achieve, except when both anti-CD4 and anti-CD8 MAbs are combined [19], with anti-CD8 MAbs probably required to control pre-committed effector T cells. Co-stimulation blockade with anti-CD40L has also been reported as less effective than anti-CD4, requiring depletion of CD8^+^ T cells even in non-sensitized animals [20], [21]. The obstacle created by sensitization in relation to tolerance induction is also evidenced by reports showing that heterologous immunity reduces the effectiveness of tolerogenic protocols in transplantation [22]. Our data shows that the anti-CD4 antibody treatment can also be beneficial to already sensitized animals. This may be due to the fact that the T effector cell frequency is significantly lower for allergens than for alloantigens and that the response to allergens is predominantly restricted to the CD4^+^ compartment. It is likely that such different outcome observed in transplantation versus allergy may be due to the induction of different tolerogenic mechanisms. In fact our preliminary data suggests that Foxp3^+^ Treg cells may play a more important role in transplantation tolerance than in tolerance induced to allergens – something that still requires further elucidation.

Our data also suggests it is likely that in sensitized mice the antibody treatment has an impact exclusively on the late response (mediated by Th2 and NKT cells), without preventing the early response mediated by mast cell degranulation in response to their surface IgE cross-linking, as the allergen-specific IgG1 and IgE titres remain high in treated mice. But even without targeting early mast cell degranulation, the MAb treatment is likely to lead to a putative long-term benefit given the importance of the Th2-mediated response for the persistence of the inflammatory changes associated with airways remodeling and chronic manifestations of the disease [4]. This issue will require confirmation in chronic models of disease.

The antigen-specificity of Treg cell-mediated tolerance has been a controversial issue [23], [24]. We show that effective tolerance induction requires the presence of the appropriate antigen at the time of CD4 blockade leading to antigen-specific tolerance. In these experiments we waited 50 days following initial sensitization to minimize the amount of antigen still present in the animal at the time of the tolerogenic treatment. These data also established that MAbs administered at day 50 (Figure 3) were not contributing significantly to the prevention of the disease by being in circulation at the time of intranasal challenge, since no beneficial effect is observed in animals treated with the same antibody dose together with an irrelevant antigen. Importantly, most previous studies addressing the putative tolerance-inducing potential of monoclonal antibodies, namely anti-CD4 [15], did not address the antigen-specificity of the phenomenon or the immune competence of treated mice.

Several MAbs have been recently used as immune modulators in a wide range of diseases, including allergy [25]. Anti-CD4 MAbs have been evaluated both in pre-clinical non-human primate models of transplantation and autoimmunity, as well as in clinical studies [13], [26], [27]. Their therapeutic effectiveness was modest, short-term, and likely to be a consequence of transient immunosuppression and not tolerance. With hindsight those unimpressive results are not surprising due to technical details related with dosing and the MAb characteristics. The clinical trials have used mouse or chimeric MAb that elicited immune responses leading to their rapid clearance [28]. In addition, most of those studies, including a clinical trial in human asthma (with the depleting anti-CD4 MAb keliximab) [13], did not take advantage of non-depleting anti-CD4 MAbs. Therefore, and as a consequence of the adverse side effects associated with depleting reagents, it was not possible to attain a neutralizing dose of anti-CD4 know to be the most effective for tolerance induction. At this time, particularly given the promising results with non-depleting anti-CD3 in early onset diabetes patients [29], the past experience of anti-CD4 in human patients should be reassessed in face of current knowledge. On this note, a new non-depleting and less-immunogenic humanized anti-CD4 MAb has been recently engineered, and its safety evaluated in human volunteers [30].

Our results suggest the specific targeting of CD4 T cells in allergic airways disease can have a potent effect in achieving long-term protection from subsequent inflammatory changes induced by the same antigens. It remains to be established whether similar effects can be achieved in chronic allergic airways disease.

## Materials and Methods

### Experimental animals

BALB/c mice were bred and maintained under specific pathogen-free facilities. Animals were sensitized, at the times described in the text, by i.p. injection of 20 µg in 2.0 mg of endotoxin-free aluminum hydroxide (Alu-gel-S, Serva, Heidelberg, Germany) of OVA or β-LG (Sigma, St Louis, USA) previously run through a DetoxyGel column (Pierce, Rockford, USA), or HDM extract (Greer, Lenoir, USA). In all experiments animals were age and sex matched.

### Ethics Statement

All experiments involving animals were approved by Direccao Geral Veterinaria (approval 018831). Mice were bred and maintained under specific pathogen free (SPF) conditions.

### Antibodies and reagents

Non-depleting anti-CD4 (YTS177) [18] and the isotype control (YKIX302) MAbs were produced in our laboratory using Integra CL1000 flasks (IBS, Chur, Switzerland), and purified from culture supernatants by 50% ammonium sulfate precipitation, dialyzed against PBS, and the purity checked by native and SDS gel electrophoresis. The hybridomas were generously provided by Professor Herman Waldmann (Oxford, UK).

### Bronchoalveolar Lavage (BAL)

Airways were washed through the trachea with 3 ml of cold PBS 1% BSA (Sigma). The BAL was centrifuged, ressuspended in PBS, and the cells counted with a hemocytometer. Differential cell counts were performed on cytospin samples stained with Giemsa-Wright (Sigma). At least 200 cells from each sample were counted, using blinded slides, to determine the relative frequency of each cell type. In addition, in some experiments eosinophilia was independently confirmed by flow cytometry using GR-1 (eBiosciences, San Diego, CA, USA), CCR3 (BD Pharmingen, San Diego, USA), and MHC-class II MAbs (produced in-house), with eosinophils identified based on the SSC/FSC profile and as the GR1^int^MHCclass II^−^CCR3^+^ cells [31].

### Quantification of immunoglobulins and cytokines

Serum titers of OVA-specific IgG1, IgG2a, and IgE were measured by ELISA using the following: IgG1 and IgG2a (SouthernBiotech, Birmingham, USA) with anti-OVA IgG1 standard from Serotec, Oxford, UK; IgE (BD-Pharmingen) with anti-OVA IgE standard from Abcam (Cambridge, UK). Cytokine titers were determined in fresh BAL and lung homogenates. Cytokine ELISAs were performed using the following kits: IL-4, IL-5 (BD-Pharmingen).

### Histology

Lungs were perfused with 4% formalin solution (Sigma), collected and sectioned. Staining was performed using hematoxilin/eosin, and periodic acid-Schiff (PAS) stain. Photographs were taken using a Leica DM2500 microscope and a Leica DFC420 camera.

### Respiratory mechanics and methacholine responsiveness

Airway responsiveness was determined 24 hours after last intranasal OVA challenge. Changes in the respiratory input impedance (Zrs) were measured using a modification of the low frequency forced-oscillation technique (LFOT) in mice anesthetized with 10 µl/g of xylazine (2 mg/ml, Ronpum, Bayer, Germany) and ketamine (40 mg/ml, Merial, Lyon, France), tracheostomized and ventilated (FlexiVent, SciReq, Montreal, Canada). Mice were hyperventilated at 450 breaths/min and Zrs was measured during periods of apnea using a 16 s signal containing 19 prime sinusoidal frequencies as described elsewhere [32]. Calculation of airway resistance (R_aw_), tissue damping (G) and tissue elastance (H) is obtained from the Zrs spectrum using FlexiVent software (SciReq). AHR was measured by exposure to an aerosol containing increasing doses of MCh (Sigma), following a baseline measurement after the delivery of a saline aerosol.

### Statistical analysis

Statistical significance was determined using the two-tailed non-parametric Mann-Whitney test and *P* values <0.05 were deemed significant (*, *P*<0.05; **, *P*<0.01; ***, *P*<0.001).

## Supporting Information

Figure S1 **Prevention of allergic sensitization with anti-CD4 MAbs.** Female BALB/c mice were sensitized with 20 µg OVA-alum i.p. and challenged with 50 µg OVA in saline i.n. on the indicated days. Some animals were treated with 1 mg anti-CD4 or an isotype control i.p. as shown. Naive mice, not subjected to any intervention, were also studied as a control group and compared with mice injected with adjuvant in the absence of antigen at the time of sensitization. (A) Cellular composition of the BAL of mice treated with anti-CD4 at the time of sensitization. (B) Cellular composition of the BAL of mice treated with anti-CD4 at the time of initial sensitization, but subjected to additional sensitization at a subsequent time. (C) Cellular composition of the BAL of sensitized mice treated with anti-CD4.
(TIF)

Figure S2 **Invasive measurement of respiratory mechanics.** Data showing the impact of anti-CD4 treatment in tissue elastance and tissue damping in response to increasing doses of inhaled MCh. These graphs complement the data on airway resistance represented in the main figures 1 to [Fig pone-0022320-g002] [Fig pone-0022320-g003] [Fig pone-0022320-g004] 5.
(TIF)

Figure S3 **Induction of AHR following i.n. exposure to the antigen.** Female BALB/c mice were sensitized with two shots of 20 µg OVA-alum i.p. 14 days apart, and challenged with 50 µg OVA in saline i.n. for three consecutive days (day 20–22), or just on day 20. Invasive measurement of respiratory mechanics was performed on the following day in presence of increasing doses of inhaled Mch. Both groups of mice, subjected to a single or three challenges with i.n. antigen, displayed similar levels of AHR (n = 6, ** *P*<0.01, *** *P*<0.001).
(TIF)

Figure S4 **Allergic airways disease in mice with different age.** Female BALB/c mice were sensitized with 20 µg OVA-alum i.p. and challenged with 50 µg OVA in saline i.n. as indicated in Figure 1. Some animals were treated with 1 mg anti-CD4 or an isotype control i.p. at the time of sensitization. Mice with 11 or 20 weeks of age were used. No significant differences between mice of different ages.
(TIF)
