# Supplementary figures and images for: Prevention of House Dust Mite Induced Allergic Airways Disease in Mice through Immune Tolerance
Source: PLoS One. 2011 Jul 26;6(7):e22320. doi: 10.1371/journal.pone.0022320 (PMC3144234; doi:10.1371/journal.pone.0022320)

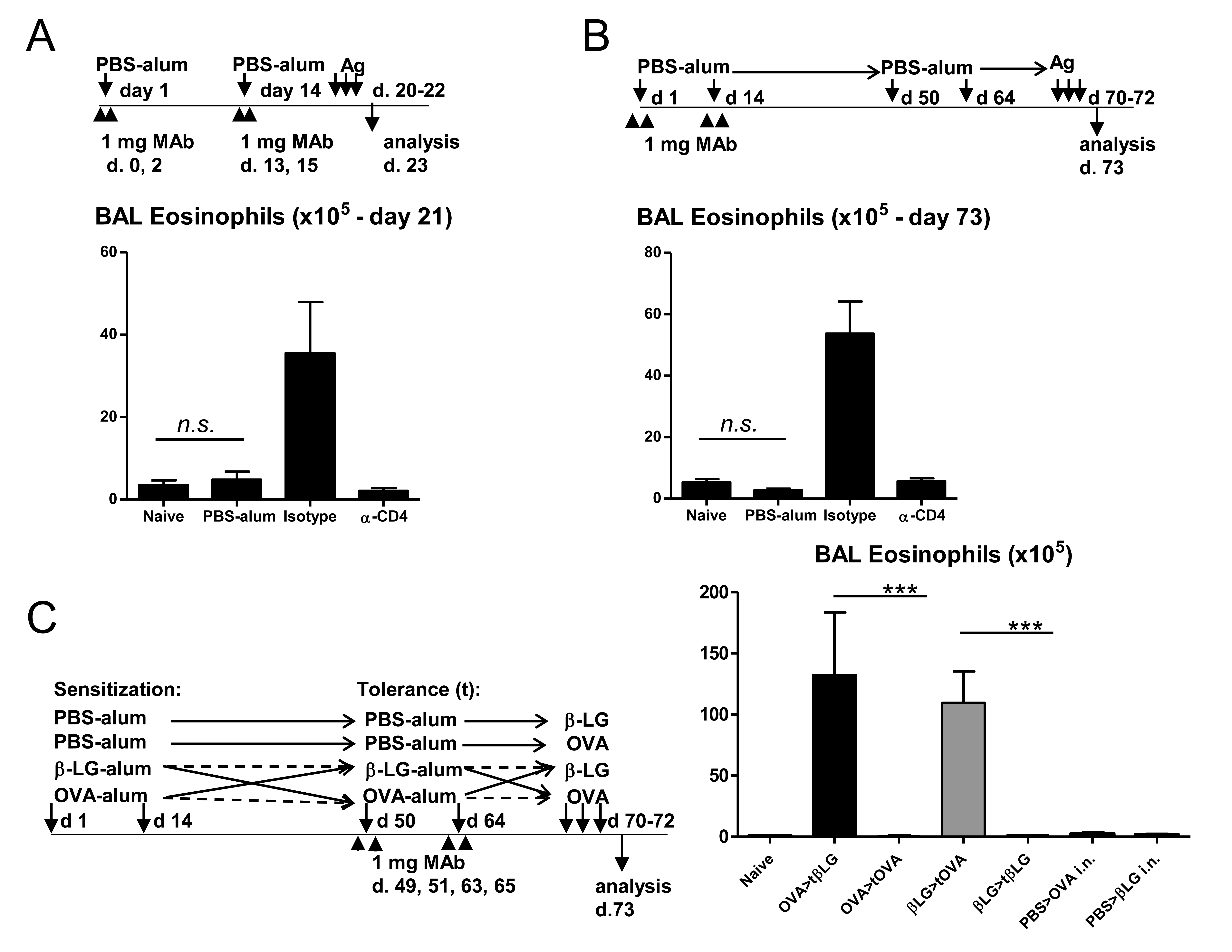

Supplement: Figure S1 — Prevention of allergic sensitization with anti-CD4 MAbs. Female BALB/c mice were sensitized with 20 µg OVA-alum i.p. and challenged with 50 µg OVA in saline i.n. on the indicated days. Some animals were treated with 1 mg anti-CD4 or an isotype control i.p. as shown. Naive mice, not subjected to any intervention, were also studied as a control group and compared with mice injected with adjuvant in the absence of antigen at the time of sensitization. (A) Cellular composition of the BAL of mice treated with anti-CD4 at the time of sensitization. (B) Cellular composition of the BAL of mice treated with anti-CD4 at the time of initial sensitization, but subjected to additional sensitization at a subsequent time. (C) Cellular composition of the BAL of sensitized mice treated with anti-CD4. (TIF) [file pone.0022320.s001.tif]

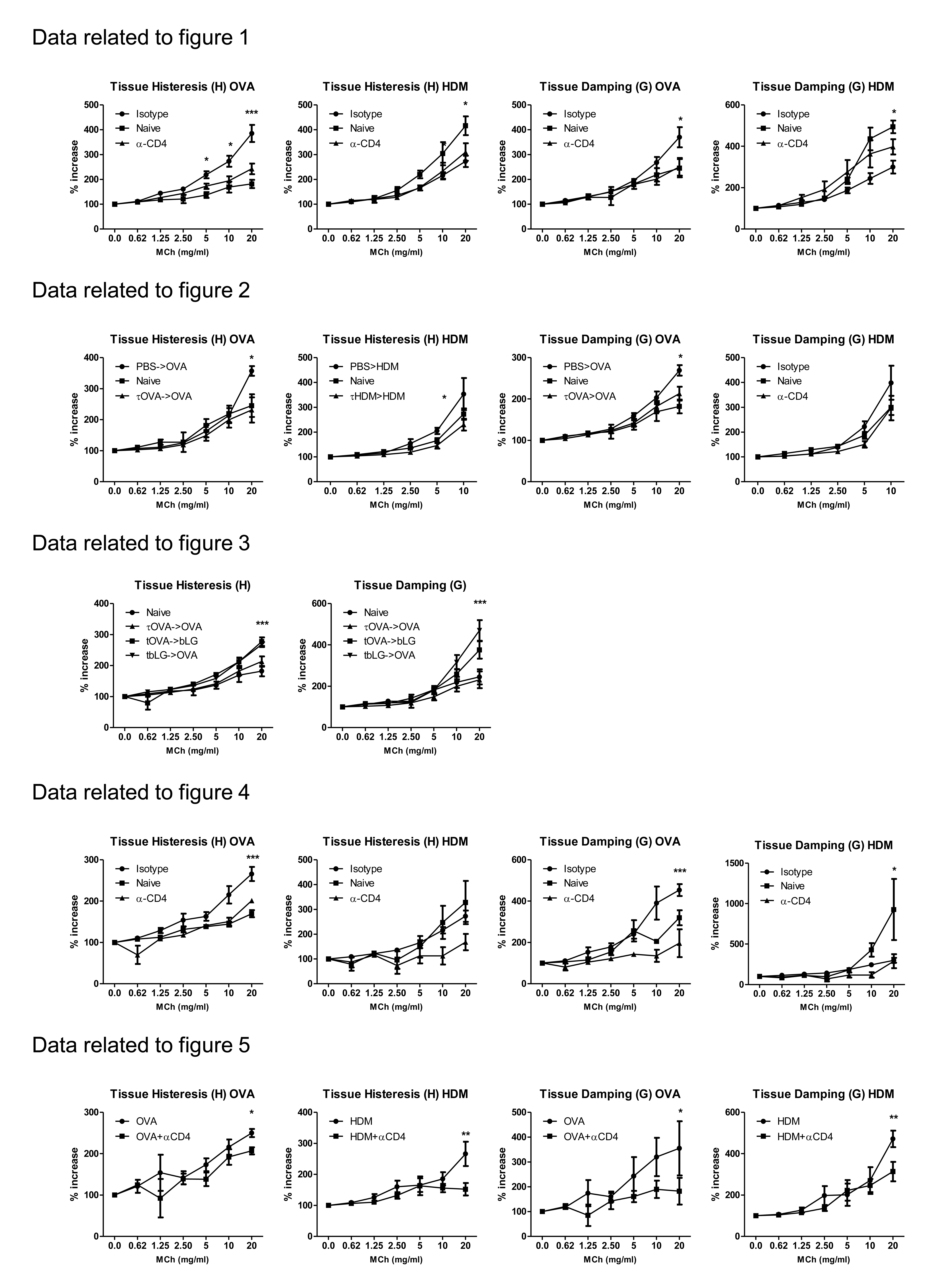

Supplement: Figure S2 — Invasive measurement of respiratory mechanics. Data showing the impact of anti-CD4 treatment in tissue elastance and tissue damping in response to increasing doses of inhaled MCh. These graphs complement the data on airway resistance represented in the main figures 1 to 5. (TIF) [file pone.0022320.s002.tif]

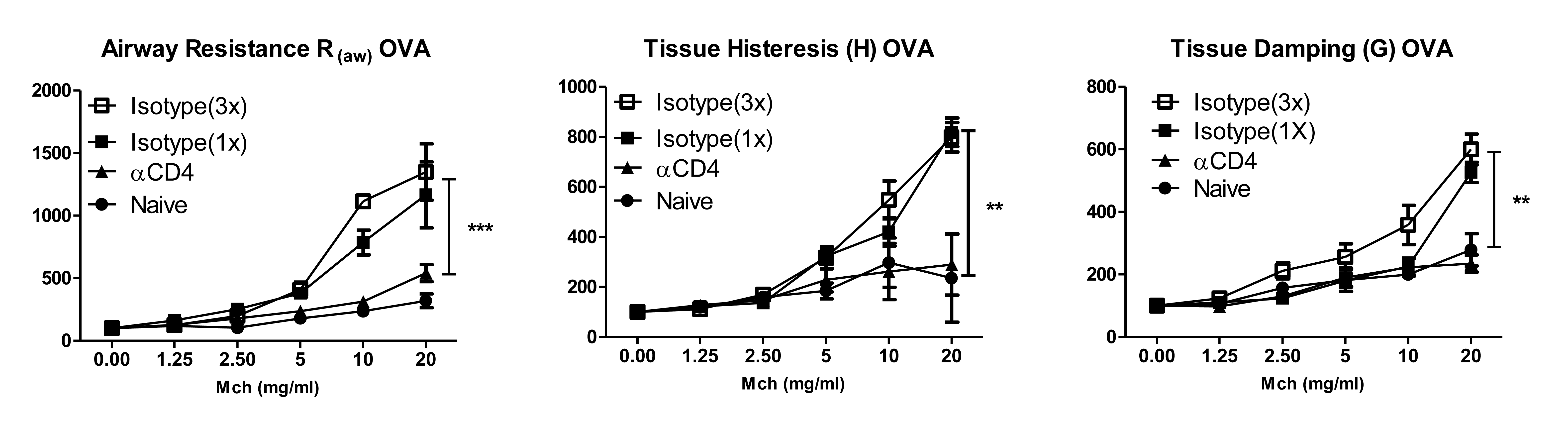

Supplement: Figure S3 — Induction of AHR following i.n. exposure to the antigen. Female BALB/c mice were sensitized with two shots of 20 µg OVA-alum i.p. 14 days apart, and challenged with 50 µg OVA in saline i.n. for three consecutive days (day 20–22), or just on day 20. Invasive measurement of respiratory mechanics was performed on the following day in presence of increasing doses of inhaled Mch. Both groups of mice, subjected to a single or three challenges with i.n. antigen, displayed similar levels of AHR (n = 6, ** P<0.01, *** P<0.001). (TIF) [file pone.0022320.s003.tif]

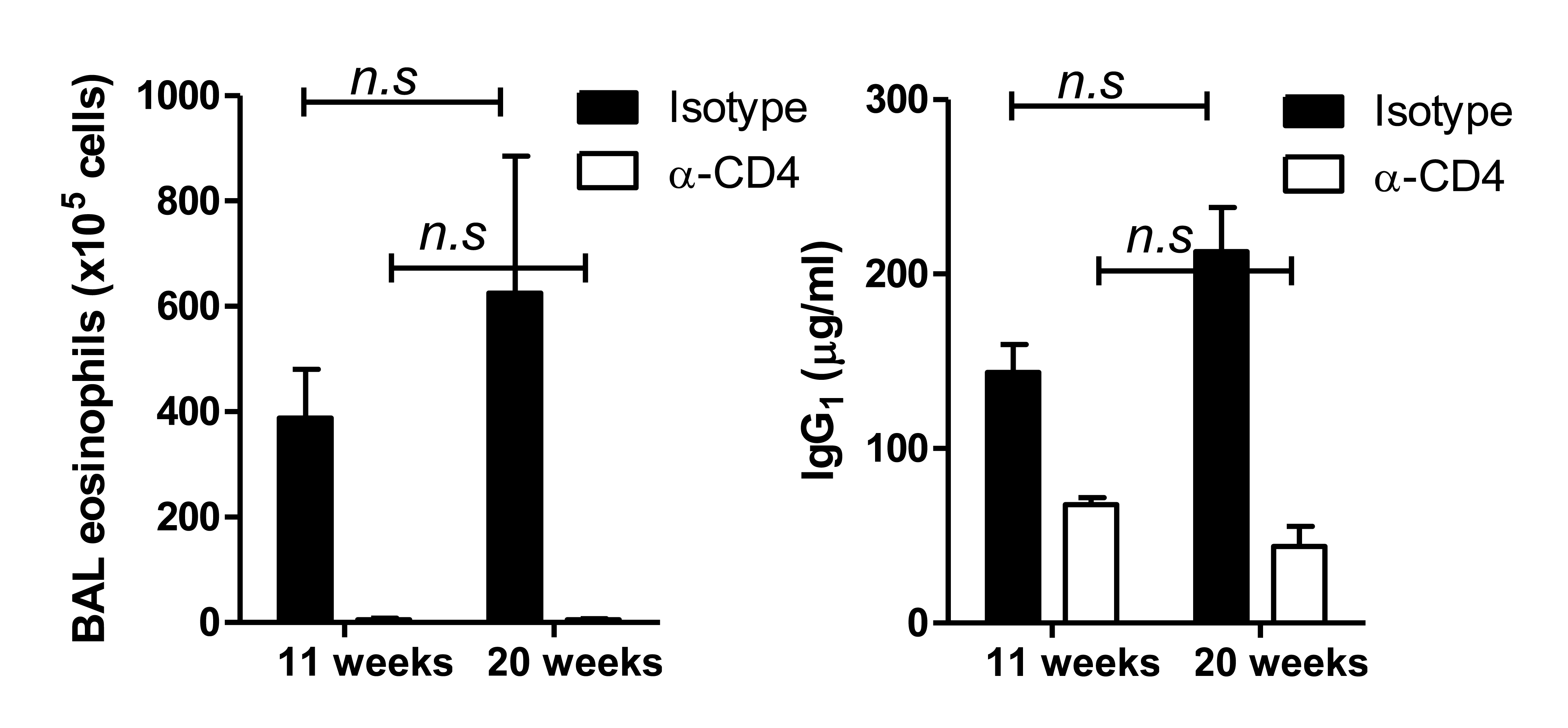

Supplement: Figure S4 — Allergic airways disease in mice with different age. Female BALB/c mice were sensitized with 20 µg OVA-alum i.p. and challenged with 50 µg OVA in saline i.n. as indicated in Figure 1. Some animals were treated with 1 mg anti-CD4 or an isotype control i.p. at the time of sensitization. Mice with 11 or 20 weeks of age were used. No significant differences between mice of different ages. (TIF) [file pone.0022320.s004.tif]
